# Multivariate Monitoring Workflow for Formulation, Fill and Finish Processes

**DOI:** 10.3390/bioengineering7020050

**Published:** 2020-06-03

**Authors:** Barbara Pretzner, Christopher Taylor, Filip Dorozinski, Michael Dekner, Andreas Liebminger, Christoph Herwig

**Affiliations:** 1 Exputec GmbH, Mariahilfer Straße 88A/1/9, 1070 Vienna, Austria; christopher.taylor@exputec.com (C.T.); christoph.herwig@tuwien.ac.at (C.H.); 2 Research Area Biochemical Engineering, Vienna University of Technology, Gumpendorferstrasse 1a, 1060 Vienna, Austria; 3 Department of Manufacturing Sciences, Takeda, 1070 Vienna, Austria; filip.dorozinski@takeda.com (F.D.); michael.dekner@takeda.com (M.D.); 4 Plasma Derived Therapies R&D, Takeda, 1070 Vienna, Austria; andreas.liebminger@takeda.com

**Keywords:** multivariate monitoring, CPV, formulation, fill finish process, data science, time-series analysis, feature extraction

## Abstract

Process monitoring is a critical task in ensuring the consistent quality of the final drug product in biopharmaceutical formulation, fill, and finish (FFF) processes. Data generated during FFF monitoring includes multiple time series and high-dimensional data, which is typically investigated in a limited way and rarely examined with multivariate data analysis (MVDA) tools to optimally distinguish between normal and abnormal observations. Data alignment, data cleaning and correct feature extraction of time series of various FFF sources are resource-intensive tasks, but nonetheless they are crucial for further data analysis. Furthermore, most commercial statistical software programs offer only nonrobust MVDA, rendering the identification of multivariate outliers error-prone. To solve this issue, we aimed to develop a novel, automated, multivariate process monitoring workflow for FFF processes, which is able to robustly identify root causes in process-relevant FFF features. We demonstrate the successful implementation of algorithms capable of data alignment and cleaning of time-series data from various FFF data sources, followed by the interconnection of the time-series data with process-relevant phase settings, thus enabling the seamless extraction of process-relevant features. This workflow allows the introduction of efficient, high-dimensional monitoring in FFF for a daily work-routine as well as for continued process verification (CPV).

## 1. Introduction

In 2011, the Food and Drug Administration (FDA) published a guideline that emphasizes the importance of undertaking continued process verification (CPV) in biopharmaceutical manufacturing as an integral and final part of the process validation lifecycle [1] within the Quality by Design (QbD) approach. CPV ensures that the product quality and process performance stays in control throughout the commercial part of the product life cycle. The core element of a CPV plan is the control and monitoring strategy of certain critical process parameters (CPPs) and critical quality attributes (CQAs), as well as the method for analyzing the collected data. The FDA stresses that the collected data should be evaluated with appropriate statistical process control technology, but leaves the selection of a concrete monitoring strategy and statistical tools to the individual developer. In the biopharmaceutical industry, most manufacturers use simple out-of-specification or univariate trending charts to show their control over their process [2,3]. While classic biopharma process segments, namely upstream and downstream processing resulting in the drug substance, typically have established CPV plans leading into validation, when it comes to advanced control technologies, formulation, fill, and finish (FFF) is often deprioritized [4]. Therefore, current and well-described CPV or monitoring plans for FFF are very difficult to find in the literature. This last unit operation is needed to turn the purified drug substance into a final dosage form, applicable for the market [5]. Freezing of the purified protein bulk, thawing of the bulk, formulation, sterile filtration, filling and on occasion lyophilization are the common steps within FFF to obtain a safe, stable, final product, ready to be transported. Although the FFF process is chemically and biologically more straightforward than a fermentation process, any variation in FFF can influence the stability, safety or final dosage form of the product [5,6].

**Figure 1:**
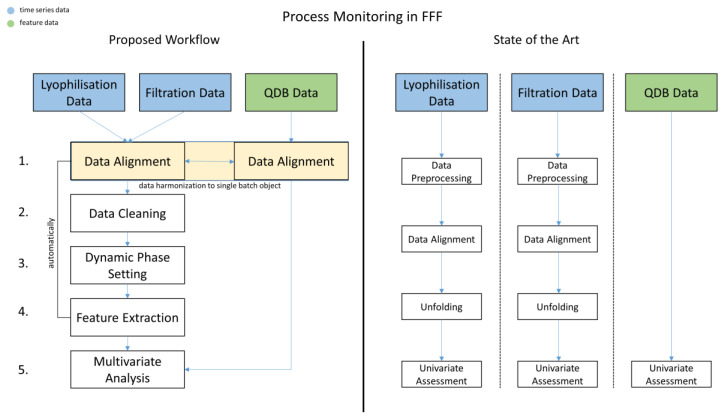
Comparison of workflows for process monitoring in formulation, fill, and finish (FFF) processes with respect to filtration, lyophilization and quality database (QDB) data. Compared to the current workflow, the proposed workflow examines the different FFF data sources in more detail, harmonizes the data sources to one single batch object, allowing the analysis of the data in a broader context, and uses their key feature data to perform multivariate analysis. All intermediate steps in the proposed workflow are automated and applicable on further FFF QDB data.

Current process monitoring strategies in biopharmaceutical FFF steps are generally limited to a univariate assessment of two distinct and separate data sources:

- single-point data (called “feature data”) from intermediates or from Quality Database (QDB) testing (see Figure 1, State of the Art, QDB Data—Univariate Assessment). Examples: lyophilization duration, sterile filtration hold time, amount of various formulation buffer ingredients, etc.
- time-series data during the individual unit operations (see Figure 1, State of the Art, Lyophilization/Filtration Data—Univariate Assessment). Examples: online measurement of product temperature over process time, online measurement of pressure during lyophilization over process time, etc.

This univariate control strategy is often insufficient to uncover root cause variation related to interactions between these data types in a multivariate space, as D.C. Montgomery already stated in 1991 [7]. Moreover, time-series data can give an additional deep insight on how the process actually performs in certain states or phases [8].

Most FFF control strategies only use very rudimentarily extracted time-series features such as the length, maximum, or minimum value of time-series data, overlooking the information hidden in time-series patterns [9,10,11]. One method to analyze a time-series in more detail, is to unfold the signal along its time-points and then use the data as input for multivariate data analysis (MVDA) [12]. In fermentation or in chemical engineering, the in-depth analysis of time series is established by analyzing the process with Fourier transform near-infrared spectroscopy and evaluating the data in conjunction with principal component analysis (PCA) and partial least squares regression [13,14]. Another method is to identify certain patterns by dividing the signal into smaller phases to enable advanced feature extraction. The advantage here is that only process-relevant information will be further analyzed with MVDA [15].

Although the identification of patterns is easily performed by the human eye, the analysis of several time-series patterns can be extremely time intensive and failure-prone, if not done automatically [9]. Today’s program languages, e.g., Python, offer a great variety of machine learning algorithms or neural networks (NN), which may be used for pattern recognition. Most of these data science tools demand a huge training dataset. In areas such as biology, genomics or biopharmaceutical manufacturing, it often occurs that the observed data holds a higher number of features p than number of observations N, also known as the p >> N problem. This high-dimensionality of the data often leads to problems when applying NN or machine learning algorithms [16,17].

Deeper insight to the process is not limited to the extraction of advanced features from the time-series data, but can be extended to the subsequent calculation of supplementary key performance indicators, which may also be subjected to MVDA [15]. Suarez-Zuluaga et al. showed in an upstream process case study how a basic, dynamic phase-setting algorithm, followed by key performance indicator extraction and MVDA accelerated the development of their process [18].

In order to establish MVDA for FFF in a CPV plan, the input dataset must be in a certain shape and must also be easily accessible, which is rarely the case in commercial manufacturing [19]. Usually, the data collected from the various monitoring equipment for individual unit operations of the FFF process is rarely stored in the same place and is often not aligned with each other, which results in a highly time-consuming task of establishing an analytical MVDA dataset.

This paper presents a novel, holistic, multivariate process monitoring strategy, combining the individual FFF data sources via time-series feature extraction using a dynamic phase-setting approach. The assessment of the power of this new method takes place on real biopharmaceutical manufacturing data and is compared to historical data evaluations that occurred based on traditional process monitoring strategies. The goal is herein to establish a multivariate, automated FFF process monitoring workflow, which uses all existing FFF data (time-series and QDB data), makes use of process-relevant time-series patterns, and is followed by robust principal component analysis (ROBPCA) to detect lots which perform atypically related to reference lots. This approach should ease and accelerate the identification of abnormal behavior within the FFF process and point to root causes for this via parameter loadings. The roadmap to realize this goal can be described in the following steps:

1. Assign the available data to the corresponding lots (see Figure 1, Proposed Workflow, Data Alignment).
2. Enhance the quality of information by reducing interference signals within the time series (see Figure 1, Proposed Workflow, Data Cleaning).
3. Identify process-relevant characteristics of the time-series pattern and leverage for further feature extraction (see Figure 1, Proposed Workflow, Dynamic Phase Setting).
4. Create an analytical data set based on the extracted features and combine with already available features (see Figure 1, Proposed Workflow, Feature Extraction).
5. Perform robust principal component analysis to assess the data set in step 6 (see Figure 1, Proposed Workflow, Multivariate Analysis).

## 2. Materials and Methods

### 2.1. Data

The analytical data set for our innovative CPV approach was derived from an industrial FFF process of a parenteral biopharmaceutical therapy, which is stored in a liquid phase. This data consisted of 58 lots and was compiled from three different FFF data sources, as shown in Table 1.

### 2.2. Software

The commercially available software inCyght^®^ Web version 2019.08 (Exputec GmbH, Vienna, Austria) and Python 3.5 (Python Software Foundation, https://www.python.org/) was used for data preprocessing, algorithm development, and multivariate data analysis. The statistical software JMP^®^ (SAS Institute, USA) was used for MVDA result comparison.

### 2.3. Statistical Methods

Robust principal component analysis (ROBPCA) [20] was performed for MVDA. In contrast to the conventional PCA [21], the ROBPCA is less influenced by outlying observations and can recover principal components of a data matrix even though its entries might be sparse to a certain extent. The ROBPCA analysis allows the evaluation of whether observations are more or less similar to each other in the multivariate space, by plotting the orthogonal distance against the score distance. A high value in the score distance means that the observation does obey the multivariate model, but certain variables have a higher or lower value compared to the average of the other observations. A high value in the orthogonal distance indicates that the observation does not follow the multivariate model and shows a different correlation. The contribution of the score and orthogonal distance of each observation allows the identification of which variables are responsible for the observed abnormality.

## 3. Results

### 3.1. Step 1: Data Alignment

The current state of data management within FFF describes the following procedure: measure various process parameters from all FFF process steps with different monitoring equipment and store the collected data on the monitoring equipment’s databases [19]. Interfaces, which enable us to harmonize and align the data between the different sources, without requiring major manual input, are rarely available. Some data harmonization usually takes place offline by manually adding certain CQA data to a quality database (QDB), which usually contains intermediate data from each FFF process step. However, the CQA data does not cover all the information, which is available within the time series.

This decentralized data management makes further multivariate data analysis effectively inaccessible. Furthermore, some systems—such as in this case the sterile filtration and lyophilization equipment—do not contextualize the collected data to any specific corresponding lot (i.e., what differentiates one lot to another is not defined within the data collection system), which makes the raw data impossible to be used for any further multivariate analysis.

In contrast to the state of the art, where every process signal is preprocessed and aligned separately (see Figure 1), we present an automated workflow, where every FFF data source is aligned and contextualized to a unique batch object within the inCyght database. By merging all available FFF data independently of their source or format into batch objects, a comprehensive insight of the data for each lot is given, which is a necessity to facilitate an automated multivariate CPV workflow.

**Figure 2:**
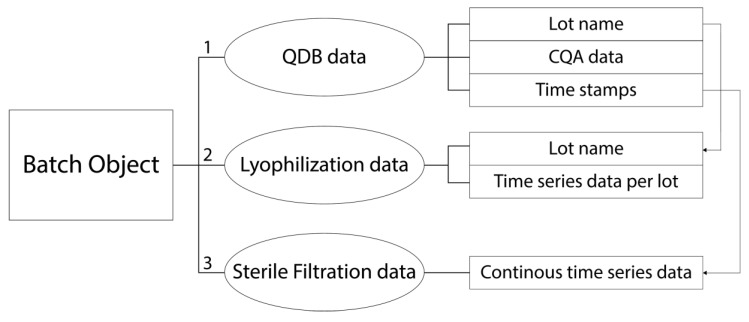
Scheme of the data alignment, showing the workflow and the individual contents of the data sources. All available data are stored in a batch object within the inCyght data base (IDB). The data alignment from various data sources to one batch object can.

To realize the harmonization of the various data origins, the data sources must be linked to each other. The most straightforward procedure is to use the lot name as linkage. However, as in the case of the sterile filtration data, no lot name was available, since the data was continuously recorded resulting in one continues time series over months. In this case we used timestamps, stored in the QDB which provided information when each lot was filtrated, to contextualize the filtration data to the batch objects. We developed a robust interface in Python, which automatically uploads all FFF data to inCyght, as shown in Figure 2.

### 3.2. Step 2: Data Cleaning

The outcome of any analysis, machine learning, or the phase-setting algorithms in the following chapters, is strongly influenced by the quality of the data [22]. Real-world data is never ideal from the analytical perspective and consists of the real signal and accompanying noise. The two main sources for noise are random noise and interference signals. Random noise is usually introduced by measurement tools such as sensors, whereas interference signals are commonly caused by equipment failure or operator error. To enhance the quality of the data, the signal-to-noise ratio (SNR) must be maximized. A common tool to enhance the SNR is the application of filters such as median or Savitzky–Golay [23]. However, the application of the wrong filter might lead to loss of data quality. Furthermore, strong interference signals might not be reduced nor removed by the usage of filters, instead leading to false-positive alerts, if not removed before the MVDA. In order to preprocess the data correctly, it is important to understand which errors may exist and to what extent they might affect the data, using domain knowledge. Different data-cleaning algorithms for each data source were developed to enhance the quality of the data accordingly, as described in the following paragraphs.

The QDB data included only CQA data and time stamps (Table 1) and was not further preprocessed. The SNR of the lyophilization data was already sufficient, where further filter application would possibly lead to information loss.

**Figure 3:**
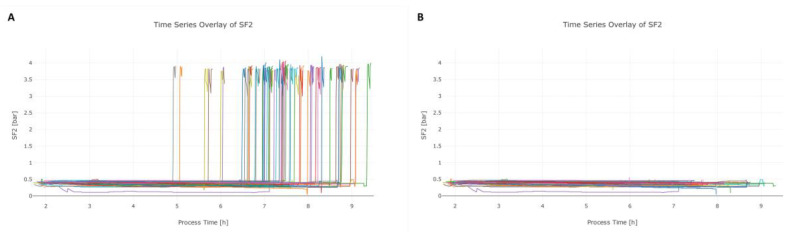
Data cleaning algorithm applied on signal SF2. The raw SF2 signals of various lots (different colors) are depicted in (**A**), whereas the high peak at the end of the process time is not relevant for monitoring and needs to be removed to enhance the performance for multivariate data analysis (MVDA). In (**B**), the corrected SF2 signal is depicted after the cleaning algorithm was applied.

Data from the sterile filtration included anomalies that did not affect the product quality, but might affect the results of the MVDA and therefore need to be removed. At the end of the filtration, the pressure increases, leading to a high peak at the end of SF2 (SF—sterile filtration), as shown in Figure 3A. Those high peaks are typical within the process, but not relevant for data analysis in this approach. Therefore, the data-cleaning algorithm was adjusted to remove the last slope from SF2, as shown in Figure 3B.

**Figure 4:**
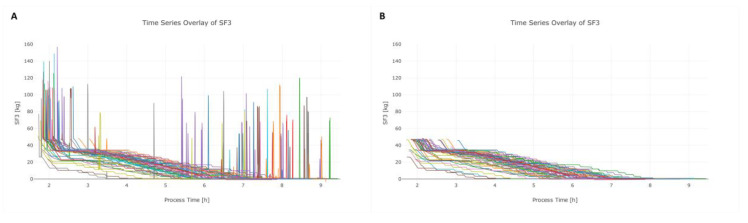
Data-cleaning algorithm applied on signal SF3. The raw SF3 signals of various lots (different colors) are depicted in (**A**), whereas the high peaks within the signal are caused due to stepping on the scale. These peaks have no further process-relevant information content and therefore need to be removed to enhance the performance for MVDA. In (**B**), the corrected SF3 signal is depicted after the cleaning algorithm was applied.

SF3 is used to track the progress of the filtration and therefore should slowly decrease over time. However, SF3 showed pronounced peaks during the process, which were identified to be caused by manufacturing personnel stepping on the scale, as shown in Figure 4A. As these events are not linked to the manufacturing process, these occurrences have no impact to the quality of the product. However, these interferences have a negative influence on the data quality. Since the overall slope in SF3 can be expected to be very low, peaks with high slopes could be easily removed by the data-cleaning algorithm, as seen in Figure 4B.

### 3.3. Step 3: Dynamic Phase Setting

#### 3.3.1. General

Time-series data are frequently used for control charts or other univariate monitoring tools, whereas only the maximum or minimum value or the length of the signal are commonly used for further analysis in the biopharmaceutical industry. Although the pattern of a time series explains the behavior of a process in greater detail, it is usually overlooked when it comes to FFF monitoring plans.

Most statistical analysis and monitoring tools require a two-dimensional data set. Since time-series data is a three-dimensional data format (batches (N) × variables (K) × time (J)), the data has to be dimensionally rearranged into a two-way matrix structure; this process is also called “unfolding”. One possibility is to unfold the data based on every single time point, resulting in N·J × K matrix. This kind of unfolding leads to a dataset with many features which are not necessarily process-relevant and might lead to a higher demand of data storage [24,25]. Another possibility is to separate the time series into phases, based on process expert knowledge, e.g., cooling or heating phases within the temperature signal. Certain features (e.g., median, mean, min, and max value) of this phase and signal can be subsequently extracted, which are more valuable for monitoring than time-dependent features. Therefore, we developed novel phase-setting algorithms, specific for FFF data, which enable automated phase setting in the data.

#### 3.3.2. Step Signal Phase Settings Algorithms

FFF data often occur as rectangular or step signals. This is caused by the stepwise adjustment of the pH, pressure, or temperature within the FFF process, which has an immediate effect on the system. This results in time-series data, most of which consist only of plateaus and sharp slopes, which may be divided algorithmically into phases.

The starting point of a slope can be determined by searching for a value within the signal, where the difference of neighboring data is above 0 in the y-direction. The end point of the slope is reached, when the change of the neighboring points in the y-direction is 0.

(1) $$slope_{start}=abs(y_{t}-y_{t+i})>0$$

(2) $$slope_{end}=abs(y_{t}-y_{t+i})=0$$

Like any other signal, the stepwise signal might contain some noise, as stated in subchapter Step 2: Data Cleaning. This could result in misidentification of slopes, since the noise could randomly indicate a gradient or plateau. In this workflow, we used several methods to tackle this problem. First, if the signal-to-noise ratio is estimable, a certain threshold for equation 1 and 2 can be chosen, instead of assuming a change of 0.

(3) $$slope_{start}=abs(y_{t}-y_{t+i})>threshold\;value$$

(4) $$slope_{end}=abs(y_{t}-y_{t+i})\leq \;threshold\;value$$

**Figure 5:**
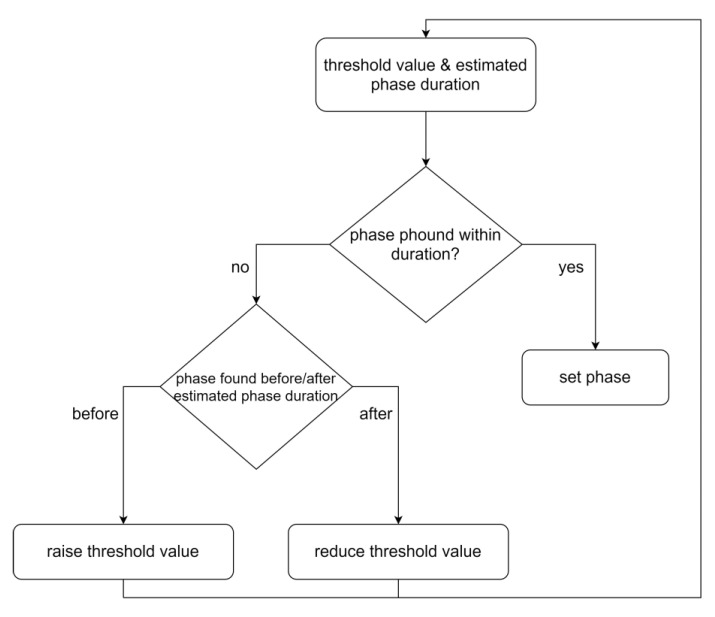
Flowchart of the step signal phase settings algorithm. The algorithm starts with an initial threshold value and an estimated phase duration range. If the algorithm detects a phase (slope or plateau) the algorithm stops and sets the phase accordingly. However, if the algorithm detects a phase before or after the estimated phase duration, the threshold value is raised or reduced, respectively. After that the algorithm starts again with the changed threshold value.

The threshold was set based on the standard deviation of the noise within the individual time-series data and was verified for practical relevance by the process experts. However, the noise might not always be estimable or unaffected by outliers, which again would result in the wrong phase setting. Such false identifications can be reduced by taking the duration of the slope and plateaus into account. If the minimum or maximum duration of the plateaus or slopes is assessable (derivable from FFF standard operation procedures or by consultation with the process expert), the current slope or plateau duration can be checked to determine if it meets the criterion of the expected length of the duration. If the criterion is not fulfilled, the current proposal for the end of the slope or plateau is discarded and the algorithm searches for the next best guess, as illustrated in Figure 5.

Based on this approach, 97% of the phases were correctly identified, which was verified by an expert from the FFF facility.

#### 3.3.3. Intertwined Phase Settings Algorithms

Not all time-series data can be divided into slopes or plateaus only, or lead to process-relevant features. Depending on the observed signal, other states might be of interest, but cannot be algorithmically divided into phases with only one input time series, since the pattern of interest results from the combination of several signals.

**Figure 6:**
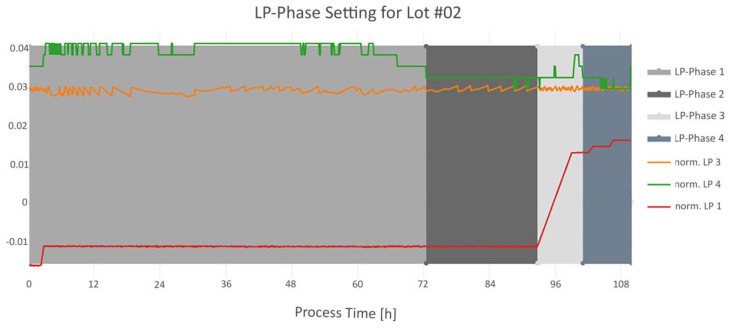
Example results for lot #2 of the intertwined phase setting method. The LP-Phases 1–4 are shown as filled rectangles in different grey colors. The normalized signal of LP3 is shown in orange, the normalized signal of LP4 is shown in green and the normalized signal of LP1 is shown in red.

Thus, a principle advantage of having centralized and aligned data is that the information from the individual time-series signals can be combined to enable process-relevant phase setting. As an example, the process engineers wanted to monitor the LP4 signal from lyophilization in more detail, by dividing the signal into four phases, to identify any deviations within the lots, as seen in Figure 6.

For this approach the LP1, LP3 and LP4 signal (LP—lyophilization) were used. The detailed phase-setting conditions for this algorithm are described in Table 2.

### 3.4. Step 4: Feature Extraction

After the phases were set, the mean, maximum, and minimum value were extracted. Furthermore, the residuals of the slope and the standard deviation of the plateau phases were calculated. Moreover, the duration of the LP Phases described in the Section 3.3.3. Intertwined Phase Settings Algorithms were extracted.

**Figure 7:**
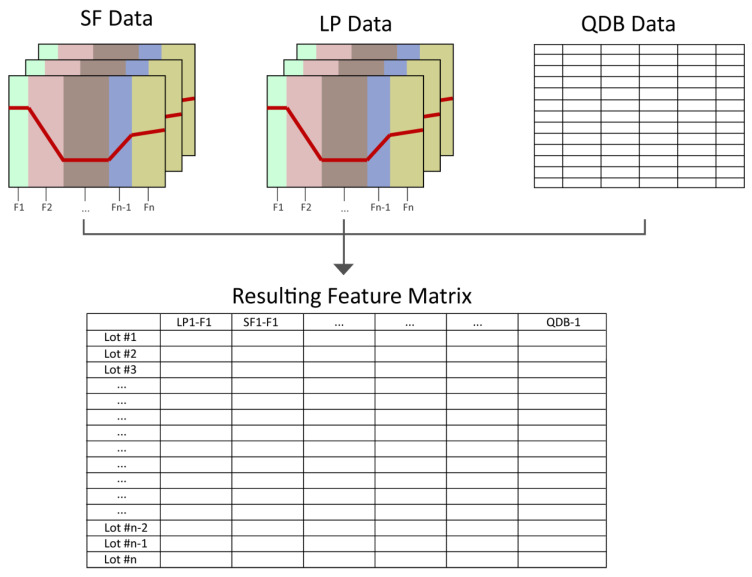
Schematic workflow of feature extraction. After the time-series data of SF and LP are separated into phases, various features are extracted (shown as F1, F2, …, Fn-1, Fn) from the data per lot and summarized in an overall feature matrix. Furthermore, the features of the QDB data are added to the feature matrix. The resulting feature matrix is ready for to be further processed by MVDA.

All extracted features, as well as the features from the QDB data, were joined in a feature matrix, as schematically shown in Figure 7. The feature extraction resulted in 130 new variables, making a total in 252 variables (including 122 QDB features). Features without variance within the lots were removed from the feature matrix, resulting in 208 features per lot in total for further robust PCA analysis (see Supplementary Materials).

### 3.5. Step 5: Multivariate Analysis

**Figure 8:**
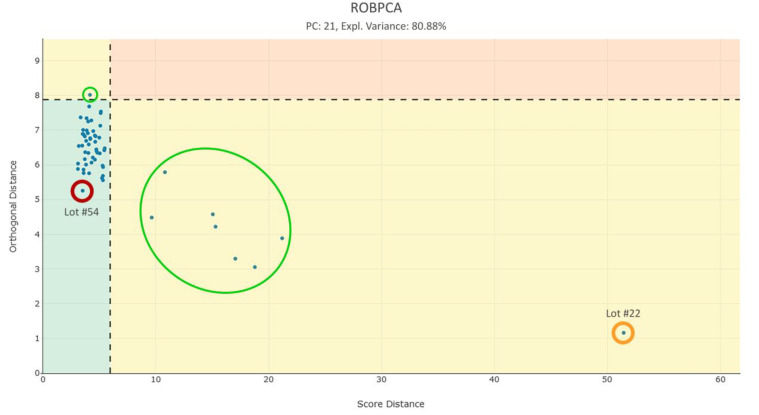
Outlier plot showing all 58 lots (blue points) with their associated scores and orthogonal distance. Additionally, the 97.5% tolerance intervals are indicated as dashed lines, which separate the plot in four differently colored quadrants. Observations in the green quadrant obey the multivariate model, whereas observations in the yellow quadrants indicate orthogonal (upper-left) or score distance (lower-right) outliers. Observations in the yellow quadrants, but close to the 97.5% TI, can be considered as moderate outliers (green circles). The red quadrant includes lots which are suspicious in both directions. Lot #22, shown as encircled in orange, is identified as most prominent outlier within the score distance. Lot #54, shown encircled in red, is closest to the model mean (0,0). No score and orthogonal outlier were observed. The robust principal component analysis (ROBPCA) was built with 21 principal components and explains 80.88% of the variance.

Since there was no CPV plan in place as of the start of this project, which would have specified any normal operating ranges for the multivariate space, we looked to identify batches that differ from the majority by certain features. This allows us to detect differences within the batches that might remain undiscovered in the univariate space. The robust PCA weighs all 252 features and 58 lots equally, making all features and lots equally important. As described and developed by M. Hubert et al., it is important to distinguish between regular and abnormal observations. Therefore a robust score distance (SDi) and an orthogonal distance (ODi) for each observation is calculated respective to the Mahalanobis distance [26,27]. This calculation is followed by plotting the distances on the y-axis and x-axis for each observation (blue points), as shown in Figure 8**.** [20]. The plot is divided by a cutoff line (black dashed line) in two yellow, one red, and one green quadrant to differentiate between outliers and normal observations. The cutoff values for the vertical and horizontal line are both 97.5% (97.5% quantile of a chi-squared distribution), again following the calculation developed by M. Hubert et al. If the lots observe the multivariate model, they are located in the green quadrant. However, if the observation is identified as an orthogonal or score outlier, it is found in the left-top yellow quadrant or right-bottom yellow quadrant, respectively. When a lot is identified as both a score and orthogonal outlier, it is located in the red/orange quadrant [20].

The majority of lots are found in the green quadrant, indicating good multivariate model for the majority of the observations. Furthermore, there are no lots, which are identified as both score and orthogonal outliers, which may be indicative of a stable process. Nonetheless, eight lots are noteworthy in their score distance, of which seven lots are moderately outlying, whereas lot #22 is the most prominent representative of this group and can be assumed to be an outlier, as seen in Figure 8.

**Figure 9:**
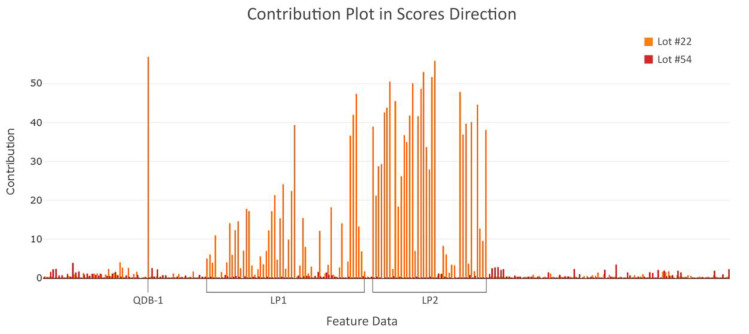
Contribution plot in the scores direction, showing which feature data (x-axis) has most impact to the contribution (y-axis) for the outlier lot and the lot closest to the center of the model. All 252 variables of lot #22 (orange bars) and lot #54 (red bars) are depicted. A strong pattern for lot #22 is identified, which is nonexistent in lot #54. Of particular note is ‘QDB-1’ and the extracted plateau features from variable LP1 and LP2, which exhibit a high contribution (≤52) and lead to the outlying score of lot #22.

In order to discover the underlying root cause for lot #22 abnormality in the score distance, the contribution plot may be applied. This plot displays the contribution of each variable to the score distance of the observation to the model plane. Variables which have a significant contribution to a lot’s score distance from the model mean have large values in the bar plot. As seen in Figure 9 a strong pattern for #22 is identified, which is not observed for lot #54 (the lot closest to the center of the proposed model). Of note, the extracted plateau features from variable LP1 and LP2 show a very striking behavior in the scores direction (see “LP1” and “LP2”, respectively, Figure 9). Additionally, a CQA feature from the QDB data exhibits the highest contribution in the score distance for lot #22, shown in Figure 9**.** as “QDB-1”. These features explain the distance of lot #22 to the model mean in the outlier plot and therefore may be prioritized for further investigation.

**Figure 10:**
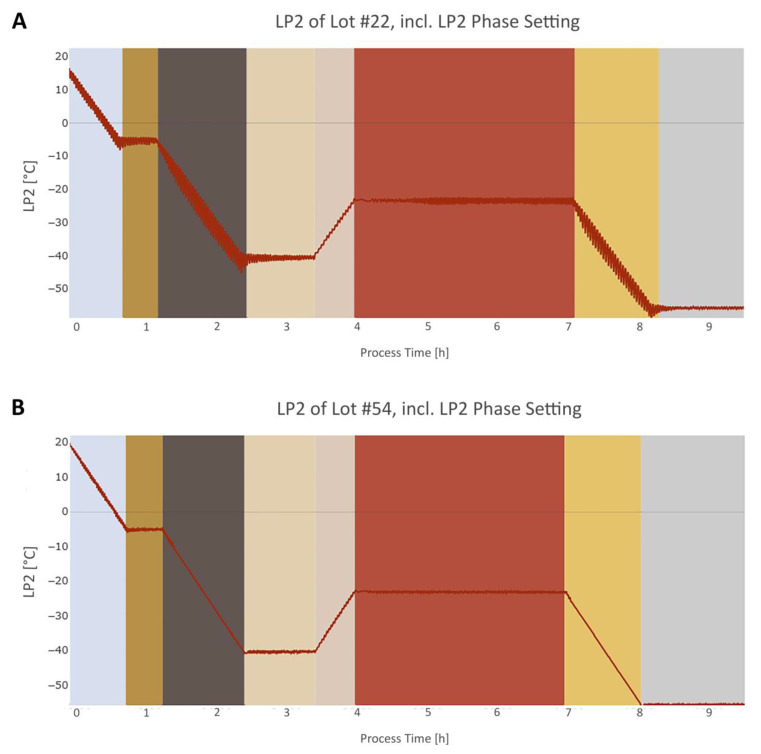
Segment of the LP2 signal (red line) over the process time of lot #22 (**A**) and lot #54 (**B**). The colored rectangles represent the individual phases for LP2, discussed in chapter 3.3.2. Step signal phase settings algorithms divide the signal into plateaus and slopes. The LP2 signal in (**A**) has a ~30% higher noise level than the LP2 signal in (**B**), which led to the high score distance in the robust PCA, as shown in Figure 8.

When comparing the LP2 signal of lot #22 with lot #54, it also becomes clear that the LP2 signal of lot #22 has a ~30% higher noise in the slopes and plateaus as the reference lot, as shown in Figure 10. Using state-of-the-art methods for monitoring in FFF, this noise had been undetected so far.

## 4. Discussion

Our presented workflow clearly demonstrates that our approach is capable of achieving the intended purpose of monitoring process-relevant abnormalities in high dimensional FFF data and pinpointing potential root causes, whereas conventional monitoring procedures would have overlooked these abnormalities. The approach represents the first workflow wherein FFF data, regardless of data type, is comprehensively and algorithmically aligned, extracted, and analyzed in a centralized monitoring system.

Data alignment is the first essential and indispensable step when carrying out multivariate analysis on data from various sources. Yet data alignment is still a big obstacle in biopharma and its solution strongly depends on the individual manufacturer [19]. After data alignment and cleaning, we reduce the data dimension by separating the time-series data into phases, where various features are extracted. It is important to note that process expert knowledge is required to decide which phases should be set and which features should be extracted to ensure that all significant production information is being monitored [28]. This is the differentiating factor to the often-used unfolding process, which does not take this process knowledge into account and thus leaves analysis purely in the hands of the data analytical tool.

Lastly, the resulting overall feature matrix requires statistical know-how to robustly identify any multivariate outlier in the data. The following subchapters discuss the applied phase setting and outlier detection in greater detail and compares them to the state of the art.

### 4.1. Phase Setting

Time-series data includes valuable information, but are often not looked at closely, since analysis is not straight forward as with tabular data records. To apply any statistical tool to the time series for analysis, the data dimension has to be reduced. Commonly, the signals are often unfolded variable- or batch-wise on every single time point [24]. This results in a dataset with many features, which are not necessarily important for process monitoring. Therefore, we present a time-series dimension reduction, where the signals are divided in phases, followed by feature extraction based on process expert knowledge.

The method for phase setting strongly depends on the shape of the signal, as well as on the sample size. In FFF time series, data from lyophilization and filtration processes from lot to lot are very similar and mainly consists of slopes and plateaus. Therefore, we were able to use fixed thresholds for phase setting, whereas the value of the thresholds can be easily evaluated with the help of process knowledge. However, one drawback of this method is, that these thresholds are very rigid and unexpected changes within the process might lead to failed phase settings. Machine learning algorithms, such has random forest could improve the robustness of our proposed phase-settings algorithms [29]. Unfortunately, as with most machine learning algorithms, success depends strongly on N to *p* ratio, that is, the quality and size of the training data set, which is rarely the case in real biopharma manufacturing processes [30].

### 4.2. Outlier Detection

The identification of abnormal observations within the monitored data is the most critical aspect of CPV. As in engineering or genetics, the data of biotechnological processes are high-dimensional. However, extreme values within the data can be easily identified by scatter or boxplots when analyzing the data in one dimension. The univariate analysis of a multivariate dataset ignores completely the orthogonal relation between the observations. Therefore, we highly recommend using MDVA for high-dimensional data to identify possible multivariate outliers. One of the most popular statistical tools is PCA, which helps to understand high-dimensional data better and to identify which variables have most effect on the variations within the data. This method tries to explain the covariance by the means of principal components (PC), which are linear combinations of the variables. The PCA can be used not only to determine latent variables in the data but also to identify outliers. Despite its popularity, one drawback of the classical PCA (CPCA) is that it is highly sensitive to outliers, resulting in a disturbed multivariate model leading to false interpretations, as shown by M. Hubert et al. and V. Todorov et al. [20,31]. Hence, we strongly recommend robust methods, as ROBPCA, when trying to identify outliers. However, the ROBPCA method uses a hard 97.5% cutoff value to distinguish between normal and abnormal observations. P. Filzmoser developed an adaptive method to estimate the cutoff value based on the data structure and sample size, which could further improve the outlier detection results of ROBPCA [32].

**Figure 11:**
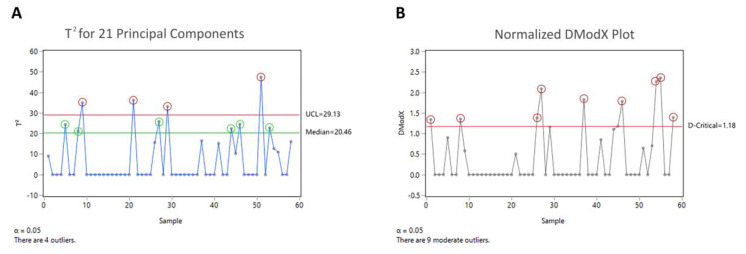
A classical principal components’ analysis (CPCA) was performed on the final feature matrix in JMP^®^. As with ROBPCA, 21 principal components were used for analysis. The Hotelling T^2^ test (**A**) and normalized DModX plot (**B**) were implemented in this software for multivariate outlier detection. The Hotelling T^2^ plot is used to identify outlying batches in the score direction, if a batch exceeds the red line, representing the 95% confidence interval (CI) of the model population, the batch can be assumed to be an outlier, whereas batches above the green line (median of the model population) can be considered as moderate outlier. The DModX plot is used to identify outlying batches in the orthogonal direction, if a batch exceeds the red line, representing the 95% CI of the model population, the batch can be assumed to be a moderate outlier. Outliers in both tests are marked as red circles.

Other statistical software such as JMP^®^ offer the possibility to analyze the data with CPCA and use the Hotelling T^2^ test and the distance to the model in the X-data (DModX) plot to identify possible outliers, as depicted in Figure 11**.**. After analyzing the final feature matrix with the Hoteling T^2^ test and normalized DModX plot four outliers in the score distance and nine outliers in the orthogonal distance were detected, respectively. Lot #22 was also identified as outlier in the Hotelling T^2^ test, but its outlyingness was not as pronounced as in ROBPCA compared to the other lots. The identification of the other outliers, identified by CPCA (see Table 3) was not reasonable, since we were not able to identify any reason for their distance-to-model-center when looking at the feature matrix. The false positive outliers might be derived from the fragile multivariate model influenced by the outlier, since CPCA is a nonrobust method.

## 5. Conclusions

We have shown that the presented multivariate FFF monitoring workflow presents a uniquely holistic centralization of all FFF data, while robustly detecting data abnormalities, which have been undiscovered with the current state-of-art methods. These alignment methods have consistently been underused, since data alignment and cleaning are resource-intensive tasks, if not done in an automated fashion. Furthermore, the classical PCA, established in most statistical software, is not ideal for outlier detection, leading to false-positive results.

We have successfully shown that the workflow is highly automatable through Python scripts and can be therefore used in CPV plans or in daily process-monitoring routines. By using automated phase-setting methods, followed by the extraction of process-relevant features and subsequent robust PCA analysis, multivariate data abnormalities can be easily identified at a glance. Once implemented, this should be easily executable by process engineers and related experts.

Once the requirements are fulfilled, such as sufficient data management and the agreement on certain phase definitions and threshold settings, process experts are able to robustly identify multivariate outliers in their FFF monitoring data with our developed algorithms in inCyght^®^. The developed algorithms could be further improved by using supervised machine learning methods for faster and more accurate threshold setting.

## Figures and Tables

**Table 1:** Overview of the quality data, sterile filtration (SF) and lyophilization (LP) data sources, including their data content, abbreviation and unit.

	Quality Data		Sterile Filtration			Lyophilization		
Data Type	Feature		Time-series			Time-series		
	Description	Abbr.	Description	Abbr.	Unit	Description	Abbr.	Unit
Monitored Output	CQA data of bulk drug substance (BDS)	CQA data	Temperature of product	SF1	(°C)	Inlet temperature	LP1	(°C)
	Time stamps of start and end of sterile filtration and lyophilization	Time stamps	Applied pressure	SF2	(bar)	Outlet temperature	LP2	(°C)
			Weight of unfiltered product	SF3	(kg)	Chamber vacuum 1	LP3	(bar)
						Chamber vacuum 2	LP4	(bar)
						Temperature of liquid nitrogen	LP5	(°C)
						Condenser pressure	LP6	(bar)
						Condenser vacuum	LP7	(bar)

**Table 2:** Condition description on how the four phases LP-Phase 1-4 were set. The phases were set accordingly to the “run order”.

Phase Name	Condition	Run Order
LP-Phase 1	Starts with the beginning of LP4 signal. Ends with the start of LP-Phase 2.	2
LP-Phase 2	First timestamp, where the difference between LP3 and LP4 is below 20%. Ends with the increasing slope of LP1.	1
LP-Phase 3	Starts with the end of LP-Phase 2. Ends when LP4 has the same value as at the beginning of LP-Phase 2, within certain time range.	3
LP-Phase 4	Starts with the end of LP-Phase 3. Ends with end of LP4.	4

**Table 3:** Comparison of CPCA and ROBPCA in terms of outlier identification.

	Score Distance		Orthogonal Distance	
	Moderate Outlier	Outlier	Moderate Outlier	Outlier
CPCA	6	4	-	9
ROBPCA	7	1	1	0

## Supplementary Materials

The feature matrix is available online at https://www.mdpi.com/2306-5354/7/2/50/s1.
